# Beyond Cure: A Scoping Review of Post-Tuberculosis Long-Term Health Outcomes

**DOI:** 10.3390/tropicalmed11070203

**Published:** 2026-07-20

**Authors:** Sonia Menon, Anthony D. Harries, Riitta A. Dlodlo, Gisèle Badoum, Mohammed F. Dogo, Olivia B. Mbitikon, Pranay Sinha, Yan Lin, Jyoti Jaju, Aung Naing Soe, Anisha Singh, Bharati Kalottee, Kobto G. Koura

**Affiliations:** 1 International Union Against Tuberculosis and Lung Disease, 75001 Paris, France; soniasimonemenon@gmail.com (S.M.); adharries@theunion.org (A.D.H.); rdlodlo@theunion.org (R.A.D.); gisele.badoum.consultant@theunion.org (G.B.); fall.dogo.consultant@theunion.org (M.F.D.); olivia.mbitikon.consultant@theunion.org (O.B.M.); psinha@bu.edu (P.S.); ylin.consultant@theunion.org (Y.L.); jyoti.jaju@theunion.org (J.J.); ansoe08@gmail.com (A.N.S.); anisha.singh@theunion.org (A.S.); bharati.kalottee@theunion.org (B.K.); 2 Epitech Research, 1160 Auderghem, Belgium; 3 Department of Clinical Research, Faculty of Infectious and Tropical Diseases, London School of Hygiene and Tropical Medicine, London WC1E 7HT, UK; 4 Unité de Formation et de Recherche en Sciences de la Santé (UFR/SDS), Université Joseph KI-ZERBO, Ouagadougou P.O. Box 7021, Burkina Faso; 5 Service de Pneumologie, Centre Hospitalier et Universitaire de Yalgado Ouédraogo (CNHU-YO), Ouagadougou P.O. Box 7022, Burkina Faso; 6 Section of Infectious Diseases, Boston University Chobanian and Avedisian School of Medicine, Boston, MA 02118, USA; 7 Boston Medical Center, Boston, MA 02118, USA; 8 Unité Mixte de Recherche MERIT (UMR261), Université Paris Cité, Institut de Recherche pour le Développement (IRD), 75006 Paris, France

**Keywords:** tuberculosis, lung health outcomes and risk factors, non-respiratory post-TB health outcomes and its risk factors

## Abstract

Background: Tuberculosis (TB) remains a leading cause of global morbidity and mortality, yet its impact extends far beyond microbiological cure. Many TB survivors experience persistent structural lung damage or functional impairment consistent with post-TB lung disease, while growing evidence highlights long-term non-respiratory health outcomes. Synthesizing evidence across lung and non- respiratory health outcomes, along with their risk factors, is critical to inform long-term TB care. Methods: We conducted a scoping review including systematic reviews reporting on post-TB long-term health outcomes. A search was performed in PubMed/MEDLINE on 27 July 2025, using terms related to “tuberculosis,” “systematic review,” “meta-analysis,” “sequelae” and “long-term health outcomes,” without language restrictions. Results: Nine systematic reviews met inclusion criteria. Most focused on pulmonary outcomes and consistently demonstrated that TB is associated with chronic airflow obstruction, reduced lung function, and an increased long-term risk of lung cancer, although residual confounding from environmental, clinical, and socioeconomic factors cannot be excluded. While younger adults are more prone to developing COPD after TB in high TB burden settings, older individuals face a higher risk of broader post-TB lung sequelae. Evidence suggested that TB survivors are at increased risk of non-respiratory complications. HIV co-infection, low CD4 counts, older age, pre-existing hepatitis, prior TB treatment, and hypoalbuminemia were associated with post-TB liver injury, while baseline hearing impairment and HIV co-infection increased the likelihood of post-TB hearing loss. TB was also linked to elevated risk of several non-pulmonary cancers, including oesophageal, cervical, hematological, pancreatic, and gastric malignancies, with the highest risk within the first year after TB diagnosis and persisting, though attenuated, in subsequent years. Conclusion: TB should be viewed as a chronic condition with enduring lung and non-respiratory health outcomes. TB survivors face increased risks of COPD, lung cancer, and a range of non-respiratory health outcomes, including hepatic and auditory complications, particularly among high-risk groups, such as those living with HIV infection, along with baseline hearing and hepatic impairment. Public health programmes must extend care beyond microbiological cure to include integrated, post-TB long-term monitoring of lung, hepatic and hearing across all ages, including malignancy surveillance, after baseline assessments to identify high-risk TB survivors. Future research should also elucidate risk factors for post-TB malignancy, and clarify the relationship between neurological, renal, and musculoskeletal sequelae and TB to inform evidence-based TB survivorship care.

## 1. Introduction

Tuberculosis (TB) remains one of the leading infectious causes of morbidity and mortality worldwide, with an estimated 10.7 million new cases and 1.23 million deaths in 2024 [1]. While advances in TB diagnosis and treatment have saved more than 83 million lives since 2000 [2], increasing evidence shows that the consequences of TB extend well beyond microbiological cure. TB survivors frequently experience long-term health effects [3], prompting recently the formal acknowledgement of post-tuberculosis lung disease at the first international symposium of Post-TB Lung Health in South Africa in 2019 [4].

Emerging data suggest that up to half of pulmonary TB survivors live with persistent structural damage or functional impairment [5]. Apart from obstructive impairment, TB-related pathology may also drive carcinogenesis through mechanisms that include chronic inflammation, immune imbalance, DNA damage, tissue remodelling, changes in gene expression, and regulation of the tumour microenvironment, all of which may induce cancer [6].

Beyond the pulmonary system, there is growing recognition of non-respiratory health outcomes among TB survivors. A recent systematic review reported high prevalence of mental health disorders (23%), musculoskeletal disability (17%), and hearing or visual loss (15% and 10%, respectively), with a greater burden in drug-resistant TB and in low-income settings [3]. Another review highlighted the spectrum of post-TB long-term health outcomes in children and adolescents, ranging from radiological residual changes after pulmonary TB, to disabling deformities after musculoskeletal and cutaneous TB and to somatic and psychosocial impairment after tuberculous meningitis [7]. Persistent undernutrition is also increasingly recognized; even after completing treatment, a substantial proportion of people who had TB—up to half in some cohorts—remain chronically underweight [8,9]. In addition, a large review revealed that post-treatment pulmonary TB is frequently followed by long-term respiratory impairment and functional limitation, with abnormal spirometry observed in approximately 60% of TB survivors [10]. Furthermore, there is growing evidence that survivors of multidrug-resistant TB (MDR-TB) exhibit greater deficits in Forced Expiratory Volume (FEV_1_ 66%), Forced Vital Capacity (FVC 76%) and a higher prevalence of mixed ventilatory impairment [11].

Recent modelling estimated that among the 8.5 million individuals surviving TB in 2019, 58 million disability-adjusted life years (DALYs) will accrue over their remaining lifetimes due to post-TB sequelae, accounting for nearly half of the overall global TB burden [12]. Although consensus-based clinical standards for post-TB lung disease have begun to emerge, these remain narrowly focused on pulmonary rehabilitation [13]. Synthesizing evidence on post-TB long-term health outcomes, lung and non-respiratory health outcomes, along with their risk factors, is essential to inform long-term care.. This review completes a structured programme of evidence syntheses examining the natural history of tuberculosis by addressing post-tuberculosis long-term health outcomes.

## 2. Materials and Methods

### 2.1. Search Strategy and PECOS Questions

A search was performed in PubMed database on 27 July 2025 using the following search string: (“tuberculosis,” AND (“sequelae,” OR “long-term outcomes”) AND (“systematic review,” OR “meta-analysis,”)). No language restrictions were applied and reference lists of included reviews were also screened to identify additional systematic reviews. We registered our protocol with OSF https://osf.io/gn74s/overview (accessed on 22 December 2024).

This scoping review was conducted in accordance with the PRISMA-ScR (Preferred Reporting Items for Systematic Reviews and Meta-Analyses Extension for Scoping Reviews) guidelines. The protocol was prospectively defined to ensure methodological transparency and reproducibility.

The primary research question guiding this scoping review was: What are the associations between TB and long term pulmonary and non-respiratory health outcomes along with their respective risk factors? To structure the review, the following PECOS question was formulated to systematically scope relevant literature:

*Population:* Adults, adolescents, and children with a history of TB (pulmonary or extra-pulmonary).
*Exposure:* Survival following TB disease and completion of treatment.
*Comparator:* Individuals without a prior history of TB.
*Outcome:* Any outcome/risk factor associated with post-TB sequelae.
*Study Design:* Systematic reviews published within the specified timeframe (2000–March 2025).

This study was designed as a scoping review of systematic reviews. Unlike an overview (umbrella review), which aims to synthesize and compare effect estimates across reviews, our objective was to map the breadth of evidence available in systematic reviews on long term health outcomes (pulmonary and non-respiratory) and the risk factors associated with post-TB sequelae, characterize the magnitude and consistency of reported associations, and identify evidence gaps. The scoping methodology was therefore considered appropriate to provide a structured mapping of the existing systematic review-level evidence [14].

### 2.2. Inclusion and Exclusion Criteria

We included systematic reviews and meta-analyses that assessed long-term outcomes among people with a history of TB. Reviews were eligible if they synthesized evidence on associations between TB and subsequent sequelae. Narrative reviews, case series, and primary studies were excluded.

### 2.3. Data Extraction, Synthesis, and Reporting

Data were independently extracted by two reviewers using a standardized form, capturing study characteristics (author, year, sample size, number of included studies, total sample size), long-term health outcomes, key quantitative findings (prevalence estimates, odds ratios, hazard ratios, mean differences, or standardized mean differences), and methodological quality as reported in the review. Any discrepancies were resolved through discussion and consensus. The scoping review adhered to PRISMA-ScR (Preferred Reporting Items for Systematic Reviews and Meta-Analyses, Extension for Scoping Reviews) guidelines [15]. The PRISMA-ScR checklist is available in the Supplementary Materials Table S1.

### 2.4. Use of Non-Stigmatizing Language

We have adopted non-stigmatizing and person-centred terminology throughout this manuscript when referring to TB and individuals affected before developing an outcome of interest, as “TB survivor”. We also use “TB disease” rather than “active TB,” except where legacy terms are needed for clarity in cited literature. This linguistic approach aims to reduce stigma, promote respect and dignity, and reflect the evolving norms in global TB research and practice [16].

## 3. Results

### 3.1. PRISMA Flow Diagram and Systematic Review Characteristics

**Figure 1:**
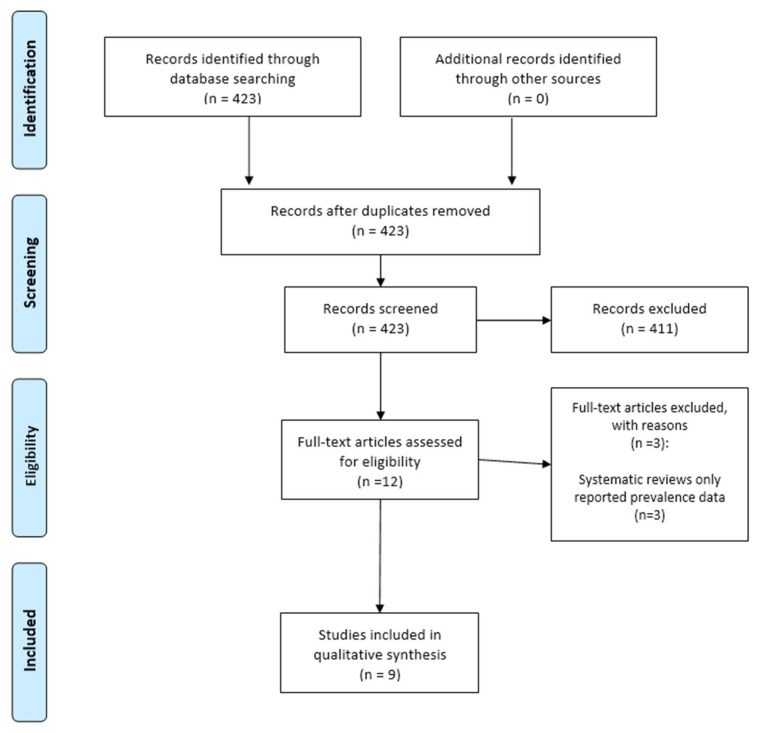
PRISMA flow diagram.

There were 423 reviews that were retrieved, of which 12 were full text screened resulting in nine eligible systematic reviews. Findings were synthesized in narrative form and organized around major domains: (1) lung health outcomes and the risk factors (2) non-respiratory TB health outcomes and the risk factors (see Figure 1 for the PRISMA flow diagram and Table 1 for the Systematic Review Characteristics).

### 3.2. Lung Health Outcomes and Risk Factors

Three systematic reviews quantified the association between TB and long-term respiratory impairment among adult TB survivors. Pulmonary TB has been shown to be associated with an increased risk of lung cancer and other malignancies across six systematic reviews.

#### 3.2.1. COPD

Across nine studies, Byrne and colleagues (2015) [17] found a pooled OR of 3.05 (95% CI 2.42–3.85) between past TB and COPD in adults over 40 years, with the strongest associations in countries with high TB burden and among never smokers and younger people. These findings suggest that TB-related respiratory damage may predispose to chronic airflow obstruction independent of traditional risk factors such as smoking. However, it was noted that residual confounding could not be excluded, particularly from passive smoking, socioeconomic disadvantage, and environmental exposures such as air pollution, which were not consistently adjusted for.

#### 3.2.2. Lung Function Impairment

One systematic review reported on risk factors for post-TB lung outcomes. Akalu et al. (2024) [18] synthesized 73 studies (31,553 participants) and identified multiple risk factors for post-TB lung sequelae, including older age (OR = 1.62, 95% CI: 1.07–2.47), previous TB treatment history (OR = 3.43; 95% CI: 2.37–4.97), smoking (OR = 1.41; 95% CI: 1.09–1.83), alcohol consumption (OR = 1.84, 95% CI: 1.04–3.25), smear-positive pulmonary TB diagnosis (OR = 3.11; 95% CI: 1.77–6.44), and the presence of radiographic evidence of pulmonary lesions at the commencement of treatment (OR = 2.04, 95% CI: 1.07–3.87). It was noted that several potentially important variables, including pre-existing respiratory conditions, baseline functional status at TB diagnosis, unemployment, limited social support, and mental health status were generally not measured or reported in the primary studies and therefore could not be assessed or adjusted for.

Ratnakumar et al. (2025) [19] reported on 7447 TB survivors within a cohort of 75,960 individuals. All studies reporting absolute values, using various levels of adjustment or standardization, showed that previous pulmonary TB had a negative effect across all spirometric values: Forced Expiratory Volume (FEV1 −0.41 L (95% CI −0.51 to −0.32, I^2^ = 90.4%), Forced Vital Capacity (FVC −0.25 L (−0.33 to −0.17, I^2^ = 80.6%), and FEV1/FVC ratio −0.37 (−0.54 to −0.19, I^2^ = 92.0%). In those studies, using reference values to derive FEV1% and FVC %, prior pulmonary TB had a pooled standardized mean difference of −0.44 (−0.60 to −0.28, I^2^ = 95.6%) and −0.33 (−0.54 to −0.13, I^2^ = 91.3%), respectively, compared with controls, suggesting that people who recover from pulmonary TB have significantly decreased lung function compared with controls, with FEV1 more affected than FVC.

#### 3.2.3. Lung Cancer

Liang et al. (2009) [20] demonstrated that prior TB was associated with a nearly two-fold increased risk of lung cancer (RR 1.8; 95% CI 1.4–2.2) even among never-smokers, an association which persisted beyond 20 years after TB diagnosis. Importantly, the relationship was not explained by active or passive smoking. Histological analyses showed a significant association with adenocarcinoma (RR 1.6; 95% CI 1.2–2.1); whereas, links with squamous and small-cell carcinoma were non-significant.

More recently, systematic reviews found similar results in people with pulmonary cancers post-TB. In a meta-analysis of 32 studies including 50,290 people with lung cancer and 846,666 controls, Abdehad et al. (2021) [21] demonstrated that prior pulmonary TB nearly doubled the risk of lung cancer (RR 2.17, 95% CI: 1.83–2.57). Elevated risks were consistent across histological subtypes, with the strongest association for squamous-cell carcinoma (RR 3.57; 95% CI 2.66–4.79).

In a systematic review of 32 observational studies, Hwang et al. (2022) [22] confirmed a significant association between prior pulmonary TB and lung cancer (OR 2.09; 95% CI 1.62–2.69), with consistent findings in studies using validated diagnostic codes (OR 2.26; 95% CI 1.29–3.94). Subgroup analyses suggested stronger associations in East Asia and the Pacific and in upper-middle-income countries, reflecting the overlapping burden of TB and lung cancer in these regions. Importantly, meta-regression revealed a heightened association in younger patients, underscoring the fact that TB-related lung carcinogenesis is not confined to older age groups.

Across 17 studies spanning high or upper-middle-income countries, Luczynski et al. (2022) [23] reported pooled standardized incidence ratio (SIRs) of 3.20 (95% CI: 2.21–4.63) for lung cancer, all highest within the first year post-TB, (SIR 4.70; 95% 1.80–12.27). In a broader analysis of 73 studies, Cabrera Sanchez et al. (2022) [24] found a hazard ratio (HR) of 1.51 (95% CI: 1.30–1.76) and OR of 1.74 (95% CI: 1.42–2.13), with the highest risk in the first 2 years post-TB (HR 5.01; 95% CI: 3.64–6.89). In the pooled analysis by latency, the adjusted hazard ratio for lung cancer diagnosis after ≥2 years of TB was 1.44 (95% CI 1.06–1.96) and was not significant at ≥7 years and ≥10 years. However, most studies did not control for passive smoking, environmental exposure, and socioeconomic status.

Sodeifian et al. (2025) [25] conducted a systematic review of 37 studies and found a significant association between prior pulmonary TB and lung cancer reporting pooled odds ratios of 2.3 (95% CI 1.4–3.8) from cohort studies and 1.9 (95% CI 1.4–2.5) from case–control studies. The association was stronger in East Asia, (OR 2.4; 95% CI 1.3–4.1), underscoring the potential benefits of targeted screening, early detection, and integrated prevention strategies in high-burden regions.

### 3.3. Non-Respiratory Post-TB Health Outcomes and Its Risk Factors

Akalu et al. (2024) [18] systematically reviewed 26 studies examining risk factors for post-TB liver injury, with 24 studies included in the meta-analyses. In HIV-infected individuals, a CD4 count < 200 cells/mm^3^ was associated with increased odds of liver injury (OR 2.03, 95% CI 1.26–3.27), as was HIV infection overall (OR 2.72; 95% CI: 1.66–4.46). Pre-existing hepatitis (OR 2.41, 1.16–6.08), prior TB treatment (OR 2.64; 95% CI: 1.22–6.67), and hypoalbuminemia (OR 2.10; 95% CI, 1.53–2.88) were also significant risk factors. Nine studies which examined risk factors associated with post-TB hearing sequelae found that baseline hearing problems (OR 1.72; 95% CI: 1.30, 2.26) and HIV infection (OR = 3.02; 95%CI: 1.96–4.64) were positively associated with post-TB hearing loss.

Luczynski et al. (2022) [23] found that prior TB was associated with an increased risk of multiple cancers, including esophageal (SIR 2.85; 95% CI 2.09–3.91), cervical (SIR 2.54; 95% CI 1.53–4.21), hematologic (RR 2.16; 95% CI 1.49–3.12), non-Hodgkin lymphoma (RR 2.31; 95% CI 1.52–3.53), Hodgkin lymphoma (RR 2.43; 95% CI 1.41–4.18), and pancreatic (RR 1.58; 95% CI 1.29–1.93) and stomach cancers (RR 1.42; 95% CI 1.15–1.75); overall cancer risk was highest in the first year post-TB (SIR 4.70; 95% CI 1.80–12.27) and remained significantly elevated up to five years thereafter.

## 4. Discussion

### 4.1. Summary of Results

Our scoping review synthesizes evidence from nine systematic reviews across diverse geographic and socioeconomic settings. Evidence indicates a link between past TB and COPD in adults over 40 years of age, with the strongest associations observed in countries with a high TB burden and among never smokers and younger individuals. These findings suggest that TB-related respiratory damage may predispose individuals to chronic airflow obstruction, independent of traditional risk factors such as smoking. Pulmonary TB has also been shown to result in lasting reductions in lung function compared with individuals without a history of TB. Survivors commonly exhibit impaired airflow and restrictive deficits, with FEV_1_ typically more affected than FVC, and although some recovery may occur early after treatment, long-term deficits remain prevalent. Risk factors associated with post-TB lung sequelae include older age, previous TB treatment, smoking, alcohol consumption, smear-positive pulmonary TB, and the presence of radiographic evidence of pulmonary lesions at the commencement of treatment.

Furthermore, a growing body of evidence suggests that TB is associated with an increased risk of lung cancer. Pooled analyses suggest an approximately two-fold elevation in risk, independent of active smoking, with associations observed across histological subtypes and stronger effects in younger patients in East Asia, the Pacific, and upper-middle-income countries. Most reviews, however, included studies that did not fully adjust for passive smoking, environmental exposures and clinical and/or socioeconomic factors, leaving residual confounding a plausible contributor. It is noteworthy, that the temporal pattern of risk remains uncertain. Some data indicate elevated risk persisting for five years or longer, while other data suggest attenuation over time, making it unclear whether the trajectory mirrors the gradual decline in lung cancer hazard observed after smoking cessation [26].

Post-TB long-term health outcomes encompass non-respiratory health outcomes. In people living with HIV, low CD4 count, pre-existing hepatitis, prior TB treatment, and hypoalbuminemia increase the risk of post-TB liver injury. Post-TB hearing loss is more likely in individuals with baseline hearing impairment or HIV co-infection. However, HIV infection itself is associated with multisystem complications, including hepatic and auditory impairment. As the included evidence did not allow comparison with people living with HIV without prior TB, these associations should be interpreted as markers of increased vulnerability rather than evidence of a specific TB–HIV interaction.

Emerging evidence also links TB to an increased risk of several malignancies beyond the lung, including oesophageal, cervical, hematologic, pancreatic, and gastric cancers, with the highest incidence observed within the first post-TB year and persisting, though attenuated, for several years thereafter.

### 4.2. Public Health Impact

The chronicity of TB, with enduring lung and non-respiratory health outcomes has substantial public health implications. At the population level, these long-term consequences translate into a substantial and under-recognized public health burden. Current global DALY estimates, including those modelled by Menzies et al. [12], simulate lifetime outcomes and attribute a considerable share of DALYs to post-TB lung disease, with nearly a third accruing 15 years or more after onset. Yet these models underestimate the true impact by failing to capture the multisystem nature of post-TB disability, its heterogeneity across subgroups, along with its sustained psychological, social and economic consequences, including stigma, and limited access to post-TB care [27].

Policy needs a shift from a narrow focus on microbiological cure to integrated, long-term lung health strategies, based upon the synergistic interactions between infectious pathogens and environmental stressors. Post-TB lung damage is an important driver of COPD risk, particularly in younger adults. COPD is now the fourth leading cause of death worldwide, with 30–40% of cases occurring in low- and middle-incomes countries [28] where TB remains endemic. In these settings, household air pollution is already a major cause of COPD [28]. When combined with rising tobacco use, alcohol consumption, previous TB treatment, older age, smear-positive TB, and presence of radiographic pulmonary lesions at the start of treatment, it may further exacerbate the risk of long-term respiratory impairment among TB survivors. This synergistic potential highlights an urgent need to embed post-TB pulmonary rehabilitation into broader national and global respiratory strategies, including tobacco and alcohol control, occupational exposure mitigation, and air pollution reduction in all TB survivors, irrespective of age. This is particularly the case for MDR-TB in whom chronic respiratory impairment is disproportionately common [11]. To this end, baseline assessment of liver function, hearing, and potentially other organ systems, including renal, musculoskeletal, and neurological status is needed to identify high-risk TB survivors and guide targeted, multi-organ post-TB care.

Evidence suggests that survivors of pulmonary TB face a significantly increased risk of lung cancer, within the first two years after TB diagnosis, with elevated risk persisting for several years thereafter. These findings underscore the importance of integrating risk-stratified surveillance and early detection strategies into post-TB care. Public health programmes should prioritize targeted screening in high-burden regions, monitor individuals with a history of pulmonary TB and scar tissue over time [29], and incorporate preventive measures such as smoking cessation and reduction in environmental exposures to mitigate long-term malignancy risk.

Post-TB non-respiratory health outcomes further necessitate a shift from short-term treatment to comprehensive survivorship care. National TB programmes would benefit from embedding structured monitoring for drug-related toxicities and providing rehabilitation and long-term social support for irreversible impairments. In particular, targeting those most vulnerable, patients with HIV co-infection and low CD4 counts, hypoalbuminaemia, or prior TB treatment through early monitoring for long-term hepatic and auditory complications would be beneficial.

Moreover, emerging evidence of TB’s chronic inflammatory and immunopathological effects, including its association also with non-pulmonary malignancies, underscores the need for integrated multidisciplinary survivorship strategies that also encompass cancer surveillance.

Finally, our review underscores the need for standardized post-treatment assessment to be part of routine post-TB care, especially for low- and middle-incomes countries. Standardized post-treatment assessments, including symptoms, spirometry interpreted using European Respiratory Society/American Thoracic Society standards with Global lung index reference ranges are increasingly feasible in LMICs. Portable, handheld devices have been shown to perform reliably compared to laboratory spirometers [30], and community-based projects in rural primary care settings demonstrate their usability when frontline health workers receive focused training [31]. Imaging, while resource-intensive, can be reserved for risk-stratified follow-up, particularly in survivors of MDR-TB, people with HIV, or those with significant residual impairment. Integrating these approaches within national TB and lung health programmes will require stepwise integration of simplified tools, targeted training, and prioritization of high-risk groups, with evidence suggesting this is feasible and impactful in high-burden, resource-limited settings [32].

### 4.3. Research Gaps

Despite growing recognition of post-TB sequelae, critical gaps remain that limit translation into clinical practice and policy which precludes the establishment of evidence-based recommendations for the management of post-TB lung disease. Future research should also prioritize the identification of post-TB health outcomes that contribute most substantially to individual disability, health system burden, and societal impact to inform risk-stratified follow-up strategies and resource allocation.

#### 4.3.1. Methodological Limitations

Existing evidence is limited by heterogeneity in study design, spirometry reference standards, and timing of assessment. Standardized definitions of post-TB lung disease are lacking, constraining comparability and hampering guideline development. Furthermore, longitudinal data beyond five years post-treatment are sparse, leaving the natural history of pulmonary and non-multisystem impairment poorly characterized. Prospective studies with harmonized outcome measures, standardized diagnostics, and internationally aligned reference values are needed to define trajectories, critical intervention windows, and inform cost-effectiveness of post-TB integrated chronic care models.

#### 4.3.2. Mechanistic Understanding

The biological mechanisms driving heterogeneous pulmonary phenotypes after TB remain poorly defined. Processes such as chronic inflammation [33], airway remodelling [33] resulting from asthma and COPD may interact with cofactors known to increase the risk of developing post-TB sequelae although the causal pathways remain unclear. Mechanistic studies rarely include MDR-TB survivors, despite their disproportionate lung impairment, limiting our ability to predict outcomes and develop targeted interventions for this high-risk population.

#### 4.3.3. TB and Cancer

The relationship between TB and malignancy, particularly lung cancer, is still poorly elucidated. Prior TB is consistently associated with elevated risk, yet causal pathways remain unclear and most studies inadequately control for confounding. The temporal relationship, including latency and stage at diagnosis, is poorly defined, limiting assessment of whether TB survivors should be included in risk-adapted screening strategies. Prospective cohorts, registry linkages, and mechanistic studies are needed to clarify latency, identify high-risk subgroups, and disentangle environmental and occupational contributions, including smoking, air pollution, silica, asbestos, and diesel exhaust.

#### 4.3.4. Non-Respiratory Health Outcomes

Although single studies have identified factors such as pre-existing hepatitis, prior TB treatment, prolonged therapy, pyrazinamide regimens, male sex, and weekly aminoglycoside use as potential contributors to post-TB hearing loss, these associations have not been systematically quantified, leaving a critical gap in understanding the determinants of ototoxicity and guiding targeted surveillance [18]. Similarly, isolated studies have suggested that higher BMI (>25), severe kyphosis (>30°), older age, spinal cord signal changes, prolonged treatment, canal encroachment (>50%), and focal motor deficits were positively associated with post-TB neurological sequelae [18], these factors have not been systematically evaluated, representing a key gap in understanding and preventing long-term neurodisability. Furthermore, the epidemiology and determinants of neurological, renal, and musculoskeletal sequelae remain poorly characterized, highlighting a need for further systematic reviews to inform evidence-based prevention and management strategies.

#### 4.3.5. Pediatric Cohorts

Pediatric prospective cohorts are needed to assess impacts on growth, neurodevelopment, and long-term organ function. Similarly, as TB survivors age, the interplay between prior TB and age-related comorbidities, such as chronic liver disease, renal impairment, and cardiovascular disease remains unclear. Systematic evaluation across age groups is essential to guide age-adapted monitoring and intervention strategies.

### 4.4. Strengths and Limitations

This review has several methodological strengths. By restricting inclusion to systematic reviews, we synthesized evidence of higher methodological rigour, enhancing the robustness and reliability of findings. Importantly, our search strategy imposed no language restrictions, broadening the evidence base and reducing selection bias, thereby increasing inclusivity across diverse regions and populations.

Nonetheless, important limitations must be acknowledged. As this scoping review synthesized evidence at the level of systematic reviews, some primary studies may have been included in more than one review, and the degree of overlap was not formally assessed. A central concern relates to confounding and bias. Many primary studies were retrospective, lacked prospective baseline measures, and did not adjust adequately for key determinants such as smoking, alcohol use, HIV, indoor air pollution, socioeconomic status, and potentially important variables, including mental health and poor social support [18], making residual confounding highly likely. Notably, passive smoking, which has a weaker association to TB, was not adjusted for [34]. Furthermore, the eligible studies generally did not adjust for socioeconomic status and environmental pollution, which have also been associated with TB and lung cancer [23]. Socio-economic status, a known risk factor for TB, is also linked to greater exposure to environmental pollutants and occupational carcinogens, and has been shown to modestly increase the risk of lung cancer and COPD even after adjusting for smoking [35,36]. Consequently, the observed associations between TB and COPD or malignancy may be partially influenced by these unmeasured or inadequately controlled confounders.

Reverse causality also represents a key limitation. Functional impairment or malignancy detected shortly after TB diagnosis may have predated the index episode and been unmasked during TB-related investigations. This concern is particularly salient for lung function studies lacking pre-treatment measurements and for cancer studies, where clinical and radiographic overlap between TB and malignancy can result in misclassification [37]. In addition, surveillance bias may inflate observed associations, as TB survivors typically undergo more frequent clinical evaluations and imaging, increasing the likelihood of detecting sequelae relative to the general population.

The quality of evidence across studies was highly variable. Several included systematic reviews also reported substantial statistical heterogeneity across primary studies, which may limit the interpretability and generalizability of pooled effect estimates. Substantial heterogeneity reflects differences in study design, spirometry reference standards, TB treatment regimens, and outcome definitions, hampering comparability and limiting the precision of pooled estimates. Further, the duration of post-TB ventilatory impairment remains uncertain. Recent longitudinal analyses suggest that these abnormalities may improve within the first few years after treatment completion, indicating that some post-TB ventilatory defects may be short-lived [38]. In our review, data on outcomes beyond 5–10 years were scant leaving the full extent of cumulative post-TB disability incompletely characterized. Residual heterogeneity likely reflects variation in case selection, study quality, timing of post-treatment lung function assessment [10], and the intrinsic biological complexity of TB, which manifests as a broad spectrum of outcomes [39].

While our review imposed no language restrictions, some systematic reviews included only English-language primary studies. This may have underrepresented data from high-burden settings. Moreover, most included studies for cancer were conducted in high-income countries, with only limited representation from the thirty highest TB burden countries, constraining global generalizability.

Finally, this scoping review relied on PubMed as the primary database, which may have resulted in the omission of relevant systematic reviews indexed exclusively in other databases, particularly those covering environmental or social science literature.

## 5. Conclusions

TB should no longer be viewed solely as an acute infectious disease but as a chronic condition with enduring pulmonary and systemic consequences. Pulmonary TB is consistently associated with reduced lung function and COPD, particularly among younger adults and in high-burden settings, while older survivors are more prone to broader post-TB lung sequelae. In addition, TB survivors face a substantially increased risk of lung cancer, most pronounced within the first two years after diagnosis. Although residual confounding cannot be entirely excluded, the consistency and biological plausibility of current evidence underscore the need for TB programmes to extend beyond microbiological cure and incorporate long-term lung health monitoring, rehabilitation, and integration with tobacco and alcohol control initiatives, for all TB survivors, irrespective of age particularly in high-TB burden settings.

As the burden of TB extends beyond the lungs, encompassing increased risks of non-pulmonary cancers and diverse non-respiratory sequelae, public health programmes must establish structured, long-term follow-up for high-risk groups. These include individuals with HIV co-infection, low CD4 counts, previous TB treatment, or those requiring hepatic or otologic monitoring. Such follow-up should integrate malignancy surveillance and chronic disease management, including targeted hepatic and auditory assessments, particularly for those with baseline hearing impairment, alongside preventive care aimed at reducing morbidity and facilitating early detection of complications.

Future research should employ standardized definitions for baseline assessments, and longitudinal study designs to clarify the biological and epidemiological mechanisms linking TB to both pulmonary and long-term non-respiratory health outcomes, including neurological, renal, musculoskeletal, and visual impairment. Identifying the key risk factors for post-TB lung disease and for lung and non-pulmonary cancers is critical to inform integrated, evidence-based, and person-centred survivorship care.

## Figures and Tables

**Table 1:** Systematic Review Characteristics included in the current scoping review.

Author of Systematic Review	Date Range of Eligible Studies	Number of Studies	Objective	Methodology Assessment	Conclusion
Byrne et al. (2015) [17]	2005–2013	9 studies (global)	Assess the association between a history of tuberculosis and the presence of COPD or chronic suppurative lung disease (bronchiectasis).	National Heart Lung and Blood Institute (NHLBI) quality assessment tool for observational cohort and cross-sectional studies (poor to good)	A history of TB was strongly associated with COPD in adults over 40 (pooled OR 3.05, 95% CI 2.42–3.85), with the strongest associations in high-incidence settings, never-smokers, and younger adults; overall, in TB-endemic areas, prior tuberculosis is closely linked to chronic respiratory disease, underscoring the need to integrate long-term lung health into TB care.
Akalu et al. (2024) [18]	From inception to 2023	73 studies (31,553 participants) 28 countries, most studies came from India, South Korea and China	Identify risk factors associated with long-term physical sequelae among TB survivors	Newcastle–Ottawa Scale (73.6% had 5–7 points, considered moderate quality study)	Older age (OR 1.62, 95% CI 1.07–2.47), previous TB treatment (OR 3.43, 95% CI 2.37–4.97), smoking (OR 1.41, 95% CI 1.09–1.83), alcohol use (OR 1.84, 95% CI 1.04–3.25), smear-positive disease (OR 3.11, 95% CI 1.77–6.44), and radiographic lung lesions (OR 2.04, 95% CI 1.07–3.87) increased the risk of post-TB lung impairment. Liver injury was associated with pre-existing hepatitis (OR 2.41, 95% CI 1.16–6.08), previous TB treatment (OR 2.64, 95% CI 1.22–6.67), hypoalbuminaemia (OR 2.10, 95% CI 1.53–2.88), HIV co-infection (OR 2.72, 95% CI 1.66–4.46), and CD4 counts < 200 mm^3^ (OR 2.03, 95% CI 1.26–3.27). Hearing loss was associated with baseline auditory impairment (OR 1.72, 95% CI 1.30–2.26) and HIV co-infection (OR 3.02, 95% CI 1.96–4.64). Post-TB respiratory, hepatic, and auditory sequelae share overlapping biological and social determinants, underscoring the need for risk-stratified, integrated survivorship care to mitigate long-term morbidity.
Ratnakumar et al. (2025) [19]	2000–2024	19 studies (75,960 individuals, 7447 with prior pulmonary TB) Altogether 47% of participants were women and many were from upper-middle-income to low-income countries	Estimate respiratory impairment after pulmonary tuberculosis disease and examine differences in ventilatory defects.	Joanna Briggs critical appraisal tool for cohort, case–control or cross-sectional studies. Score varied between 50 and 100% with 75% or more representing high quality	TB was consistently associated with reduced lung function across all spirometric values (FEV1 −0.41 L, FVC −0.25 L, FEV1/FVC −0.37), with pooled analyses confirming significantly lower FEV1% and FVC% compared with controls; overall, people who recover from TB demonstrate mixed obstructive and restrictive impairment, predominantly airflow obstruction.
Liang et al. (2009) [20]	1966–2009	41 studies (China, Taiwan, Korea, EU, North America)	Assess the relationship between preexisting TB and lung cancer risk	No quality assessment. No substantial evidence of publication bias was found overall, including in analyses restricted to never-smokers and for adenocarcinoma lung cancer (all Egger’s and Begg’s test *p* > 0.05)	Prior TB was associated with a significantly increased risk of lung cancer, including among never-smokers (RR 1.8, 95% CI 1.4–2.2) and after controlling for passive smoking (RR 2.9, 95% CI 1.6–5.3), with the elevated risk persisting for over 20 years and being particularly significant for adenocarcinoma (RR 1.6, 95% CI 1.2–2.1), supporting a direct link between TB and lung cancer independent of tobacco exposure
Abdeahad et al. (2022) [21]	1987–2021	32 studies (EU, China, Singapore, Taiwan, Korea	Assess the association between previous pulmonary TB infection and lung cancer risk	Newcastle–Ottawa scale, 5 studies had a score under 5, considered of poor quality	Prior active pulmonary TB significantly increases lung cancer risk overall (RR 2.17, 95% CI 1.83–2.57) and across histological types, adenocarcinoma (RR 2.61, 95% CI 1.71–3.98), small-cell (RR 2.12, 95% CI 1.54–2.91), squamous-cell (RR 3.57, 95% CI 2.66–4.79), and other types (RR 2.75, 95% CI 2.30–3.28), highlighting the need for post-TB lung cancer screening and extended follow-up.
Hwang et al. (2022) [22]	1190–2020	32 studies (East Asia and Pacific, Europe, Central Asia, and North America regions	Appraise observational studies reporting an association between pulmonary TB and lung cancer.	Newcastle–Ottawa scale, score 7–9 (good quality)	A history of pulmonary TB is significantly associated with lung cancer (OR 2.09, 95% CI 1.62–2.69), with stronger associations in studies using robust TB diagnostics (OR 2.26, 95% CI 1.29–3.94), in countries with medium/high TB burden, East Asia–Pacific, and upper-middle-income regions, and particularly in younger patients, highlighting TB as an independent risk factor for lung cancer.
Luczynski et al. (2022) [23]	1980–2021	17 studies (all studies were from high or upper-middle-income countries	Primary objectives were to estimate the pooled risk of all and site-specific malignancies in people with TB compared to the general population or suitable controls. The secondary objective was to describe the pooled risk of cancer at different time points following TB diagnosis.	Risk of bias in non-randomized studies of interventions. 12 studies had a serious risk of bias	Compared with controls, individuals with prior tuberculosis had a higher pooled standardized incidence ratio for all cancers (1.62, 95% CI 1.35–1.93) and for lung cancer (3.20, 95% CI 2.21–4.63), with the highest excess risk observed within the first year after diagnosis and persisting beyond five years. TB is associated with an increased risk of both pulmonary and extrapulmonary cancers. There is a need for targeted research to inform screening and early detection strategies and for clinicians to maintain a high index of suspicion for malignancy following TB diagnosis.
Cabrera-Sanchez et al. (2022) [24]	1980–2021	73 studies (sample size numbers unknown)	Explore whether TB is a risk factor for subsequent lung cancer	Newcastle–Ottawa Scale. Most studies had moderate to high risk of bias. The Grading of Recommendations, Assessment, Development and Evaluation assessment of the evidence reveals overall low certainty for cohort studies and very low certainty for case–control studies	Pooled analyses demonstrated an increased risk of lung cancer among individuals with prior tuberculosis, independent of age and smoking. Quantitative estimates showed a hazard ratio (HR) of 1.51 (95% CI 1.30–1.76; I^2^ = 81%) and an odds ratio (OR) of 1.74 (95% CI 1.42–2.13; I^2^ = 59%). The risk was highest within the first two years following TB diagnosis (HR 5.01, 95% CI 3.64–6.89) and declined thereafter. There was limited adjustment for confounders such as passive smoking, environmental exposures, and socioeconomic status. The temporal association between TB and subsequent lung cancer underscores the need for prospective studies to clarify causality and identify high-risk groups for targeted surveillance and prevention.
Sodeifian et al. (2025) [25]	From inception to 2024	37 studies (130,774 TB participants; non- exposed group 948,656 participants)	Provide a comprehensive understanding of the relationship between lung cancer and a history of TB	JBI critical appraisal checklist, the majority of studies were rated as high quality	A consistent association between prior pulmonary TB and subsequent lung cancer was observed across study designs (OR: 2.3; 95% CI 1.4–3.8) in cohort studies and (OR: 1.9: 95% CI 1.4–2.5) in case–control studies, and the strongest associations seen in East Asia (OR 2.4, 95% CI 1.3–4.1). These findings provide robust evidence that pulmonary TB increases the long-term risk of lung cancer, highlighting the need for integrated public health strategies incorporating targeted screening, early detection, and smoking cessation, particularly in high-burden settings.

## Data Availability

The data that support the findings of the study are available from one of the first authors (K.G.K.) upon reasonable request.

## Supplementary Materials

The following supporting information can be downloaded at: https://www.mdpi.com/article/10.3390/tropicalmed11070203/s1, Table S1: Preferred Reporting Items for Systematic reviews and Meta-Analyses extension for Scoping Reviews (PRISMA-ScR) Checklist – Post-tuberculosis long-term health outcomes [15].
